# “You can create a little bit more closure in your own story when someone really connects with it”: exploring how involvement in youth peer support work can promote peer development

**DOI:** 10.1186/s13033-023-00608-4

**Published:** 2023-10-24

**Authors:** Tanya Halsall, Mardi Daley, Lisa D. Hawke, Jo Henderson, Kimberly Matheson

**Affiliations:** 1 University of Ottawa Institute of Mental Health Research at The Royal, 1145 Carling Avenue, Ottawa, ON K1Z 7K4 Canada; 2 Department of Neuroscience, Carleton University, 1125 Colonel By Drive, Ottawa, ON K1S 5B6 Canada; 3 LOFT Community Services, 721 Bloor St. W Suite 103, Toronto, ON M6G 1L5 Canada; 4 Centre for Addiction and Mental Health, 1000 Queen St W, Toronto, ON M6J 1H4 Canada; 5 Department of Psychiatry, University of Toronto, 250 College Street, Toronto, ON M5T 1L8 Canada

**Keywords:** Realist evaluation, PAR—participatory action research, Qualitative research, Youth mental health and substance use, Peer support

## Abstract

**Background:**

Peer support relates to the provision of social/emotional support that is delivered by individuals with lived experience of a key characteristic that is shared with clients. Although the main objective of peer support is to enhance client outcomes, through their involvement, peers derive a secondary benefit to their own personal development. This study applied a hybrid participatory-realist approach to identify what works, for whom, why and in what circumstances within the LOFT Transitional Age Youth (TAY) peer services. This paper presents findings related to the processes and possible benefits of being involved in peer work for the peer supporters themselves.

**Methods:**

Semi-structured interviews and focus groups were completed with peer and non-peer staff from the TAY program. A qualitative analysis applied a retroductive approach that involved both inductive and deductive processes to identify relevant themes.

**Results:**

Four program theories and one over-arching context were identified through the analyses. Program theories were related to: (1) enhancing self-efficacy and self-determination through peer involvement in program design, (2) increasing peer resiliency and self-care through effective supervision, (3) developing professional skills and opportunities for career advancement through peer practice and (4) overcoming stigma through the recognition of the value of peer lived experience.

**Conclusions:**

Peer practice holds significant potential for the enhancement of the mental health system as well as to increase our understanding of stigma. The findings from this study offer critical new insights into the dynamics of how professional peer practice can support the personal development of youth peers and how programming can be intentionally designed to enhance these benefits.

## Introduction

Peer support has been defined as social and/or emotional support that is offered from the vantage point of an individual with lived experience of a mental health, social, or medical challenge that is delivered by mutual agreement to a service user that shares similar challenges to support self-determined personal growth [1]. As an approach that integrates youth perspective to enhance service delivery, youth peer support aligns with an increasing recognition of the value of patient-oriented research and emphasis on children’s rights as translated within youth engagement practices in mental health services [2–4]. Therefore, youth peer support represents a model that may be valuable to enhance research and practice while advancing social justice and equity within youth-oriented services.

Although there is limited existing research that identifies how and why youth peer support might be effective [5–7], there is emerging evidence that involvement in peer roles may be beneficial for peers themselves [8–10]. This study applied a hybrid participatory-realist evaluation to examine peer services for transitional age youth with complex mental health and substance use challenges. We present findings that describe how involvement in peer support roles might be beneficial for peers. This study utilizes a qualitative design that includes semi-structured interviews with Transitional Age Youth (TAY) staff.

### Peer support influence on peers

Youth peer support models have become increasingly popular within mental health practice [11, 12]. Although the target of peer support is mainly focused on enhancing health outcomes for program beneficiaries, it has been recognized that through their involvement, peers derive a secondary benefit [1]. In adult populations, research has identified that peers can benefit from a range of positive outcomes, including enhanced self-acceptance, sense of purpose, self-esteem, self-efficacy, confidence, coping skills, social skills, social connections, professional skills, employment opportunities and quality of life [13, 14]. Of key importance, involvement in peer roles may have a positive influence on self-stigma [1, 13]. Although benefits have been identified with adult peer providers, there is a need to identify whether these positive influences are also related to youth peer support roles [14]. Effects and processes related to young people may differ as they are navigating a different developmental period and transitional stage of life.

Despite the possible advantages to peer support roles, it is important to acknowledge that peer work can also expose peers to risks. There is substantial evidence that youth peer roles often lack clarity and this creates constant challenges for peers to navigate [8, 10, 12]. Youth peers have also identified that their roles can often place them in stressful situations that have the potential to be triggering [10, 14]. Finally, youth peer support workers are often exposed to stigmatizing attitudes and differential treatment in the workplace [8]. Given the complex and intersecting impacts of peer work for the peers themselves, it is critical to examine how programs and service can be adapted to best support youth peers in their work and personal development, while mitigating the potential risks [8].

Involving youth peers in research design is the most effective way to identify how peer work can enhance client wellbeing [11]. Youth participatory evaluation is an approach to evaluation that engages young people as decision-makers within the design, implementation and dissemination of findings [15]. Involving peers within evaluation research offers an added benefit, in that they bring insight from the lens of both practitioner and client perspectives [16].

### Purpose

This study applied a hybrid participatory-realist approach to identify what works, for whom, why and in what circumstances within the LOFT TAY peer services. The focus of this paper is on the experiences and specific processes of influence that affect peers through their involvement as peer supporters and how this work might benefit them. Data was collected through semi-structured interviews with TAY staff.

## Method

We worked in partnership with the TAY program at LOFT Community Services to implement this study. The TAY program serves young people aged 16–25 and includes a number of programs and services, including social support, group drop-ins, case management, mental health supports, navigation, campus-based services, housing and peer support. Many of the TAY program clients are coping with challenges related to mental health, substance use, chronic health issues or a dual diagnosis (developmental disability combined with mental health challenges). Some clients are also at risk of homelessness.

It is important to note that all peer staff who are recruited and trained within the TAY program are previous clients of the TAY program, therefore, they have first-hand experience of being both a program beneficiary and service provider. Since peers are recruited from programs they have participated in as a client, they begin with a strong understanding of the program style and content and they are familiar with other staff when they are hired. Peer training includes an introduction to TAY policies and values, documentation training, and shadowing other peers and programs at the beginning of their role. Peers participate in a monthly community of practice with other peers to discuss topics related to day-to-day work, role clarity, self-care, job expectations, boundaries and other aspects of peer work. Peers are also invited to participate in organization-wide staff trainings, such as in Applied Suicide Intervention Skills Training (ASIST) suicide prevention training and dialectical behaviour therapy skill development.

The TAY program also includes comprehensive supervision for peers that involves regular meetings to provide opportunities for de-briefing, reflection, and one-to-one coaching delivered by experienced supervisors [17]. In particular, supervision also supports peer decision-making with respect to self-disclosure and the negotiation of boundaries. Supervisors typically have a background in social work and peers can also access peer supervision on a monthly basis so that they can learn from colleagues experience and recommendations. LOFT will send peers for additional outside supervision if it is perceived that it would be beneficial to peers, or supervisory needs are beyond the scope of practice for internal supervisors. In addition, due to the number of recent fatal overdoses and suicide, they facilitated grief counselling outside of the agency. LOFT also offers a comprehensive training program for peers beginning their employment within the TAY program that includes an introduction to professional workplace skills, training in self-care and opportunities to participate in formalized and/or credentialed training [17].

This study combines methods from both realist and participatory evaluation design, allowing both theory and lived experience to guide key decisions within the research process (see [16, 18]. Participatory evaluation with young people advances social justice [15, 19]. This is achieved by integrating the voices of youth with lived experience, that is, young people with first-hand knowledge of the issues and contexts of concern. This integrates a dimension of critical insight that can only be achieved through lived reality. Social justice is enhanced as the youth participation can be applied to benefit the individuals involved, the system and the broader population of interest [20–23]. There are many models that have been used to inform participatory evaluation and research with youth [24, 25]. We applied a comprehensive design that involved close collaboration with one lead peer and involvement of the broader peer staff in all key decisions (see Halsall, 2021 for more detail).

Realist evaluation was designed to examine complex systems by taking contextual features, human interpretations and dynamic influences into account [26]. This approach applies mixed methods to examine process and outcomes to identify how a program works [27, 28]. Within realist evaluation, a key method involves the development of Context-Mechanism-Outcome-Configurations (CMOC) to test theoretical hypotheses about how and why a program might be effective [27]. Within a CMOC, “context” is used to represent the general conditions that must be present in order for a program to operate effectively. These conditions might be created by service provider or client characteristics, they may describe cultural or social norms or they could also represent environmental features or other systems that are required to set the foundation for the program functioning. The term “mechanism” represents the causal influences that interact between participant experiential awareness and program activities that serve to generate desired impacts [29]. Finally, the “outcome” of a CMOC equation represents the related benefits that are derived through participation in a program. We would like to note that peers are not typically the stated beneficiary of a program. Therefore, this study was not originally designed to examine how the program affects them. However, through our inductive analysis, we identified that peers experience their own personal benefits through their work and subsequently, we developed CMOCs that capture the potential processes through which this might occur.

As described in Halsall and colleagues [16], an all-staff workshop was held to launch the study and included an introduction to the study purpose, general evaluation principles and an exploratory discussion to examine current issues and research questions of interest to the TAY staff. This workshop was held during a TAY staff meeting that included peer staff, non-peer staff (e.g. Case managers), students and administration from the program. After the workshop, an initial semi-structured focus group and individual interviews were facilitated with TAY staff (peers N = 8; other staff N = 12; supervisors N = 3). These interviews focused on developing program theories with respect to how peers support client recovery. Examples of questions included: Please describe how peer support services work? What kinds of characteristics are important to be a successful peer support worker? What kinds of support do youth peer support workers provide?

Interview recordings were analyzed in QSR NVivo using a retroductive approach that involved both inductive and deductive processes (see [30, 31], including an exploratory thematic analysis [32]. Initial interviews were divided among three separate coders. A second and third round of coding was completed by two coders and then all codes were reviewed to come to consensus. One of the coders was also a peer from the TAY program (MD). Through these analyses, we identified context, mechanism and outcome codes as well as three initial CMOCs that were related with peer development and recovery (see [16] Halsall, 2021).

CMOCs were refined through a second round of interviews with peer staff only (N = 9). TH and MD developed the revised interview guides. Interview guide questions included the following: Have you had opportunities to provide input into program/service development (please describe)? In your experience within supervision, what has been the key personal insights you have developed (and have they been helpful)? How, if at all, has your experience as a peer influenced your career development? (for a full description, see [16]. Finally, the peer-focused theories were validated through peer feedback. Peer CMOCs were shared with the TAY peer staff (N = 6) through an online presentation and discussion and a Zoom poll was used to collect feedback with respect to how much they agreed with each program theory (see Table 1 for results).

**Table 1:** Validation results from peer staff feedback on program theories

	Over-arching Context	CMOC1	CMOC2	CMOC3	CMOC4	Over-arching Outcome
Level of agreement						
Strongly agreed	5 (83)	3 (50)	5 (83)	5 (83)	6 (100)	6 (100)
Agreed	1 (17)	3 (50)	1 (17)	1 (17)	0 (0)	0 (0)
Neutral	0 (0)	0 (0)	0 (0)	0 (0)	0 (0)	0 (0)
Disagreed	0 (0)	0 (0)	0 (0)	0 (0)	0 (0)	0 (0)
Strongly disagreed	0 (0)	0 (0)	0 (0)	0 (0)	0 (0)	0 (0)

This study was approved by the Royal Ottawa Health Care Group Research Ethics Board (REB# 2019007). All procedures were performed in accordance with relevant guidelines and informed consent was received from all participants. Participant numbers were created for each participant (P# for peers and S# for supervisors). Participants were a diverse group that was representative of the clientele of LOFT, including perspectives from newcomer, gender-diverse and racialized groups.

## Results

**Fig. 1:**
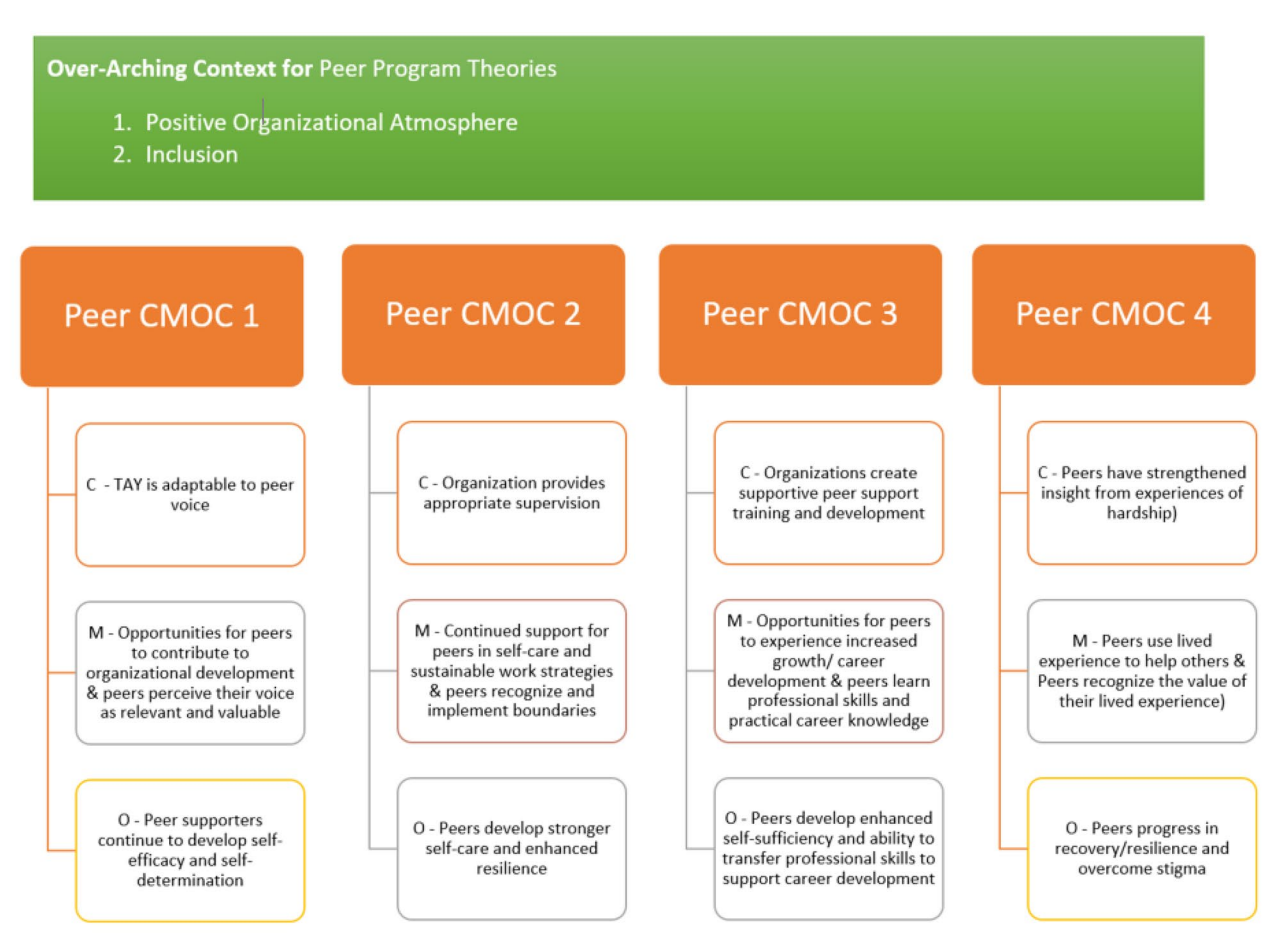
Diagram presenting over-arching context and the four program theories that were derived from the retroductive qualitative analysis

Through our analysis, we identified one over-arching context (positive organizational atmosphere/inclusion), and four individual program theories that are presented in Fig. 1.

### Positive organizational atmosphere / inclusion

Creating a positive organizational atmosphere where peers felt included and respected was a foundational aspect of the TAY program. Peers felt that their perspectives were considered to be important and they felt comfortable at LOFT. “LOFT has been like another home, I guess, in a way, for me.... Like [I] have a lot of positive experiences wrapped up in my experiences with LOFT.” (P1) “At LOFT, everyone does have a pretty high respect level for peers.” (P2).

Peers observed that the organization and staff perceive the peer role as meaningful and valuable and recognized that the peer contribution was instrumental to supporting client recovery. They felt this was demonstrated in the ways the organization moved beyond superficial inclusion. “I think that I’ve stopped being treated like a very helpful client and more like what I imagine a peer would be treated like.” (P3) “I’m not just here for lived service or to just be like ‘I’m a peer and this looks good for this organization.‘” (P4) This approach is very far removed from a tokenistic approach where peers are used in superficial ways and their roles are used to convey the appearance that youth are being engaged in the organization yet they are not involved in meaningful ways as described below.

‘Cause you can have peers come on, and they can just you know, be like a knick-knack or like a table dressing. But like here it’s like, you have to actually, if you want them to succeed, if you want them to move forward, you have to listen and you have to treat their opinion with some validity. (P5)

The four individual program theories are discussed in the following sections.

### Peer CMOC1: developing self-efficacy and self-determination

The first program theory illustrates a process whereby the peers are actively engaged in program design and decision-making. Through this approach, the peers recognize their perspective as valuable and experience strengthened self-efficacy and self-determination through this process of engagement. “When I say something, it’s respected and my input is something that’s important.” (P3) “LOFT is very open to trying suggestions and you know, seeing the value in what peers see and… bring to the table.” (P5)

Well it’s made me feel like I have a purpose in this role… It’s helped my confidence a lot to just think that I have ideas that are worth sharing to other people. Like, what I say is important and people like it. Very simple concepts but can really affect how someone moves forward in their life. (P4)

Experiencing this recognition supported peer investment in the organization and dedication to the program.

To see like you’re impacting the way that the organization is going to work with peers and is going to teach and train peers in the future…it makes you feel like you have more stake in the agency. (P2)

Peers described circumstances where their views were important for moving several aspects of programming forward, including creating and planning of events and activities to engage with clients in non-traditional ways. “So we have like submissions and we rented out a gallery space, did curation… I was one of the main organizers of [LOFT Art show]. It was a team effort, but yeah, I was one of the people that came up with the idea in the first place.” (P1) “So, like taking clients to go see a play, you know? Or to go see a dance performance, like Toronto International Film Festival was a big one and it was cool. What was nice about it was, I didn’t have to do the outreach for that and I just came up with the idea.” (P4).

Peers were involved in research and evaluation and they described how they applied their lived experience to inform research question selection and framing.

When we were doing research, now it’s like, how do we make the right questions? If we don’t frame the role properly then we can’t ask the right questions, you know? So, I think reflecting on my story to inform better practices and better research and program planning is another way.” (P4).

Peers described how LOFT supervisors encouraged their development and helped them to recognize their own leadership potential. “They saw my leadership before I did.” (P4)

She saw the raw potential in me too. That I had the skills to do that and when she started TAY, like 6 years ago, she came right to me and asked, you should do [peer work]. When I was living in the houses, I was always the first person to get their chore done and then have their room clean, so I started to help other people do that and really build like a cohesive environment in the house… She was kind of teaching me all through those years how to be in this line of work. (P5)

Being receptive to peer input and ideas while simultaneously providing opportunities for peers to engage in program planning contributes to peer self-efficacy and personal leadership development.

### Peer CMOC2: enhancing self-care and resilience

The second program theory describes the key contribution of supervision within peer development. Within this process, appropriate supervision supports peer resilience through the development of self-care and sustainable work strategies. An important part of this is building relationships with peers so that they are comfortable communicating and seeking support.

The places, like TAY included, but also like other workplaces where I’ve seen really positive peer relationships, they’ve had supervisors that have invested a lot of time into building strong relationships with those peers, you know? (P4)

Effective supervision not only builds comfort in the peer’s ability to approach their supervisor, it is a mechanism that encourages supervisors to appreciate each peer’s unique strengths, needs and work styles.

[Supervision] gives the supervisor a view on the peer to say ‘Oh, this is what happens when they are not feeling well.‘ It’s a two-way street of observing behaviors.” (P4).

Both peers and supervisors emphasized the importance of getting to know the peers. “Just being able to have that dialogue with someone who knows your habits even just from spending time with you on the workforce or checking on you, as a person, can really help.” (P4) They agree that supervision can be used to support peer development and help them to continue forward in their work.

And so you have to work with those strengths and help like build them up and reflect those back to [the peer] and say ‘Here’s really strong and here are those areas of growth’… One of my joys of being a supervisor to the peers is seeing how they feel like a sigh of relief when they can share that they’re having a difficult time and that like they’re still doing an amazing job (S3).

Peers described how supervisors helped them to problem solve issues that they encountered in their roles. They felt it was helpful when they listened first and then helped to guide them second - without offering them solutions, just perspectives.

My favorite part of supervision is, is that I get to kind of unload everything, and have my supervisor, just listen. But then also, continue listening to let me problem solve… They don’t give me the answer. … I feel like, my supervisor adds, in her own personal perspective. (P6)

Common challenges that arose for peers were related to difficulties with role clarity and boundaries with clients. Peers felt that it was not always easy to manage the boundary of professionalism while also supporting a more human connection for clients and that supervision helped them to navigate this position. “Working with supervisors who are very human has given me insight into how I can be myself more in the organization…. just being human, but also being professional at the same time.” (P2)

Any time that those kind of big barriers come up, like when I started working here for example and the client that got a little bit too close… [my supervisor] kind of gave me strategies on building those walls. (P7)

Through supervision, peers were able to work through some of their own challenges and achieve further personal development. Part of this was recognizing that peers might need more care as they transition out of formal services and also having flexibility so that peers can take time off when they need it. “Even being able to mention to a boss… that I’m starting to feel a bit worse and I’m just trying to work through some personal stuff and he’ll try and give me some skills to work through it.” (P7) Overall, peers stated that the responsibility for creating an environment conducive to vulnerable conversations rested with supervisors and management.

But I do think it’s a responsibility of peer supervision, to recognize that for many folks, they may have just left their case manager. They may just be making that transition and so things could be harder and more, on the surface, more vulnerable for them. So having that extra care and support and coaching and mentorship is important. (S3)

Beyond the benefits offered through supervision, the peer roles also served to promote general professional development as described in CMOC3.

### Peer CMOC3: Professional skill development and career advancement

The third program theory is focused on professional skill development and career advancement for peers. One benefit of the peer practice at TAY is that peers learn practical professional skills through their roles. These skills serve them in their peer work and are transferrable to other employment contexts. Some of these skills were closely related to social work and therapy, but others were general professional skills like time management and communication skills. “We’re grooming [peers] and supporting them to become a consistent and reliable employee in this profession.” (S3) “I think working as a peer has significantly improved my work prospects and I think that learning and growing soft skills is probably the most important thing you can do.” (P1).

Peers were also able to articulate the value of skills transference from their peer roles.

It’s a good practice, to learn how to be concise you know? It’s a very technical thing, but I think self-disclosure helps us communicate better. Knowing what needs to be said and what doesn’t need to be said at all times… I think that’s a very powerful skill to have in life. (P4)

Peers described the skills that they learned, as well as the career opportunities that they were able to access through their involvement at TAY. “A peer just left who was there for like three or four years and they had a decent amount of vertical job mobility in the agency.” (P2)

I think that it has like totally changed my perspective on myself and my skills. Like, I never would have gone into any sort of like social-work work without having this peer position kind of fall into my lap a little bit. Being a client and then having this job offer, totally changed my whole skill set… So, like I now have an interest in doing counselling and doing therapy. Maybe not exactly what I expected from my life, but it kind of opens some things up for me that I wouldn’t have done on my own. (P1)

Another important aspect of this function of the TAY peer practice is that their target population (youth experiencing multiple mental health challenges) often experience struggles entering the job market and need some supports to not only enter the market, but sustain their work. Having meaningful employment is a key piece to moving out of unhealthy behaviours and contexts.

[We] give them that opportunity of stability and having a job and learning skills… You get trapped in it if you don’t have the next step. Like, there’s so many people that I’ve known who have quit [substances] and kind of get their life together, but then they won’t find employment, and then what do you do now, well my life goes to drugs. You need that there in place or you’re just going to fall back in….it’s the kind of stepping stool to allow people who were clients or do have issues in their life that have caused barriers to getting to stable jobs or give them the experience they need to get a stable job, so it’s like, it works both ways. It helps the staff and clients and it also helps the actual peer. (P5)

The benefits of their role not only had impacts on their practical work skills but also fundamentally influenced their personal identity and recovery journey as described in the final program theory.

### Peer CMOC4: overcoming stigma and progressing through recovery

Peers using their lived experiences in peer practice helps them to recognize the value of that experience and subsequently supports them in overcoming related stigma. Peers bring a range of lived experiences with them to inform their personal practice, including experiences related to addictions, homelessness, human trafficking and mental health challenges. They described the hardships related with these experiences, and how they now felt thankful that they could use this insight to help others. “To turn vulnerability into something that, helps [peers] feel more empowered… It can kind of be more energizing at times.” (P6)

It sucked at the time. Like it really was, you know, it was just hell. But having that happen to me really has been the best. It has really given me a lot of dimensions that a normal person wouldn’t have. So I am I am grateful in a way that I have had these experiences that, you know, sucked. (P5)

I think you can create a little bit more closure in your own story when someone really connects with it, and feels that like it’s been impacting their lives. And it makes me feel like, the life that you’ve gone through, it wasn’t for nothing. (P7)

Peers felt that helping others supported them in their own development. “[Peers are] in the journey of becoming an adult, of becoming whoever they want to be, and they’re doing it in a way that allows them to help others.” (P1) Some of the peers talked about how they wanted to give back because of the help that they received as clients. They described how their mentors helped them to move forward in their development and that many clients coming to TAY need this kind of support in order to recognize their own potential.

So where I see myself now is just trying to make space for other people to meet their potential. I knew this was my potential. I knew I wanted to be a leader. That’s due to other people believing in me… One person behind me, I can accomplish the world. But a lot of people you see in services, don’t have one person behind them. This is the difference. (P4)

Peers talked about the benefits they experienced through contributing and how they felt rewarded for helping others. “It’s a give and take, like, as much as they feel I’m helping them, they are helping me remember why I do this. Why I get up every day, and choose to live as opposed to die.” (P6) “Now it’s come full circle. Where, now I’m helping others and giving them the opportunity to get out of those difficulties and trauma… cause it’s a really difficult, ladder out of a dark hole.” (P5).

Ultimately, peers recognized that the trait or experience they may have been stigmatized for allows them to help others in profound and meaningful ways, and in turn helps them to overcome the negative effects of stigma.

I have talked to a couple people that I work with and they have clients that they would be interested in me doing peer work with… That’s just reassuring that they have respect for what I’m going to be doing. And, it does, I think, degrade some of that internalized stigma that I think a lot of us probably have. (P2)

Helping others also helps peers to feel that they are involved in meaningful work that also contributes to their overall wellbeing. “It gives me like a sense of self-purpose, I guess, just in terms of like I am using my knowledge.” (P2).

## Discussion

This research was designed to examine what works, for whom, why and in what circumstances within the LOFT-TAY peer services. The findings describe the factors and processes that influence peer development through their involvement as peer supporters. Four program theories and one over-arching context were derived from the analyses. Although previous research has begun to identify the potential benefits of peer work for peers themselves [1, 13], this study extends this work by identifying potential mechanisms that support these outcomes.

### Evidence supporting program theories

There is existing evidence that aligns with the program theories that were identified. In other research, peers have reported developing confidence through their roles [9, 10, 14] and that this has contributed to their sense of purpose [14] and confidence beyond their peer roles [10]. These findings lend support to the first theory that describes an interaction whereby TAY created opportunities to support peer participation in program development and this process supports enhanced peer self-efficacy and self-determination (CMOC 1).

There is also evidence that substantiates the second program theory that predicates the role of supervision in enhancing peer resiliency and self-care (CMOC 2). Peers have previously identified the key importance of supervision [9, 10, 14]. In particular, peer supervision has been identified as effective for mitigating job-related stress, particularly related to managing conflict with non-peer staff and overcoming vulnerability when they were new to the position [8]. Similarly, in the present study peers and supervisors emphasized the key supports that should be provided within supervision as well as the strengths that can be developed, such as capacity for self-care and enhanced resilience.

In terms of the third program theory that highlights the process of peer skill development and career progression (CMOC3), previous studies have found that peers experience skill development in a broad range of areas, including improved employment and organisational skills, a general sense of improved ability to work with young people and families, and increased social, interpersonal and communication skills, including, but not limited to appropriate self-disclosure [10]. Further, peers have suggested that their work in peer roles have supported their entrance into the job market [10] and increased their employability [9, 10].

The last program theory proposes that the peer role offers the opportunity for peers to find value in their lived experience and that this recognition liberates them from the stress of related stigma and supports enhanced recovery (CMOC4). This aspect is an intrinsic component of all peer practice and may have the most profound impact on peer wellbeing and development. This underlying process may be foundational to why peer practice can be effective. In our research examining program theories related to TAY client recovery, we found that peers’ positive self-regard was key to supporting clients in recognizing themselves as a part of a positive social group that shared a stigmatized characteristic and that this recognition helped them to overcome the negative effects of stigma [18]. Therefore, this process, may create a reciprocal effect that benefits both peers and clients within peer practice.

Previous research has identified that through their work, peers recognize that their lived experience has significant value. “I never knew I could turn negative things in my life into a career that can give hope to others” (8, p. 5; 14). Peers report that their experience helped to normalize mental health issues, “Now I can help other people and let them know it’s okay and, like, there is nothing to be ashamed of” [10, 14, p. 911]. Finally, in line with the person-centred and strengths-based philosophy of peer practice [33], previous research has recognized the importance of inclusion and engagement in youth peer work [8, 10, 14]. This offers support to the over-arching context of a positive and inclusive organizational atmosphere.

### Theoretical applications

**Fig. 2:**
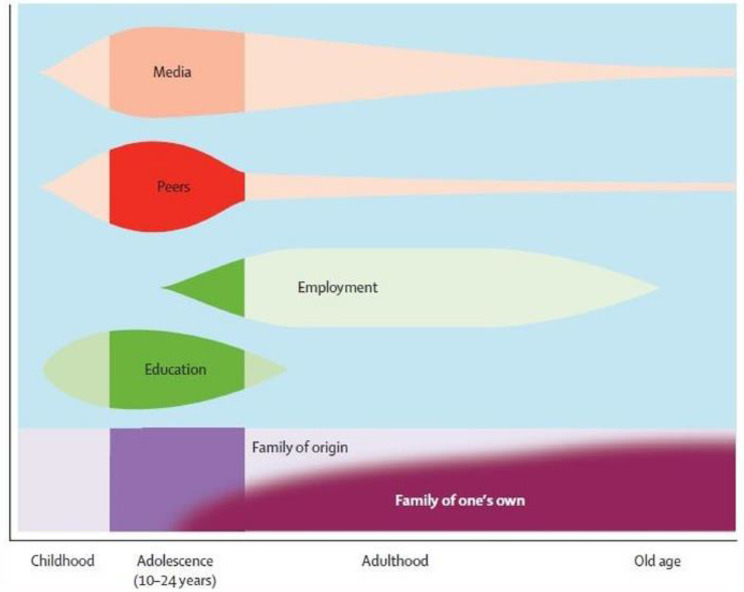
Social contexts that influence development across the lifespan. The level of peer influence increases during the stage of adolescence. The influence of the employment context increases in later adolescence and is maintained through adulthood toward old age. Reprinted from Patton, G. C., Sawyer, S. M., Santelli, J. S., Ross, D. A., Afifi, R., Allen, N. B., … Viner, R. M. [2016]. Our future: a Lancet commission on adolescent health and wellbeing. The Lancet, 387[10,036], 2423–2478 with permission from Elsevier

These program theories were initially developed from the inductive qualitative analysis, so there was no specific theory being tested through their development, however, the third theory, which describes peer skill development and career progression, aligns well with the bioecological model [34]. The bioecological model describes the interaction process between a developing individual and their surrounding context and it highlights the influential role that social determinants play in the promotion of healthy development. Involvement in peer support represents a significant developmental context for transitional aged youth. The peer role helps young people to launch a sustainable job within a positive and supportive environment that can act as an initiation into a successful career. This opportunity can serve to support their autonomy and general independence as they move into adulthood. Receiving youth peer support services and then transitioning to a peer role takes advantage of two significant social determinants within key developmental windows (see Fig. 2). First, peer services help to develop healthy social connections to other peers. Later, moving into a peer role aligns with a transition away from the importance of peer influences toward a positive employment context, which has the potential to impact them through the trajectory of their adulthood.

### Future research and recommendations

These program theories were developed inductively through an exploratory process and they should be examined and further tested within other youth peer program contexts. Peer practice has been shown to have both positive and negative effects on peers [8, 10, 12], therefore, these theories should be further explored in order to maximize benefits for peers and mitigate risks through the development of tailored peer programs.

If these theories are validated in future research, they should be considered and applied in youth peer program design and training. By extension, the program theories can be used to develop program logic models and evaluation measurement strategies to identify areas for improvement and to examine challenges experienced by peer staff in their roles. Further, these findings suggest that it may be useful to re-conceptualize peer programming to more formally recognize that peers are a secondary program beneficiary. If peer programming is used to sustain and promote peer recovery, it serves the dual function of promoting wellness for peers while contributing to client health promotion. Young people coping with mental health challenges benefit significantly from the advantages offered from engagement in meaningful professional advocacy work [35]. Envisioning peer support as a health promotion strategy for peers moves the perspective beyond the clinical setting toward a more systemic approach that can be used to support increased equity and more sustainable outcomes for peers.

These findings also highlight the importance of and benefits related to supervision, however, there is a need to better understand how to strengthen supervision supports in order to benefit clients and peers. Relevant research questions that might shed light on this practice include examining how to balance client and peer needs within supervision? In addition, what privacy conflicts can arise when one supervisor provides services to a client and supervision to a peer? How can they be mitigated? These lines of investigation can help to shape supervision practice to enhance peer support effectiveness for both clients and peers.

Recognizing that the stage of adolescence and young adulthood is short, organizations should be mindful to develop progressive opportunities for youth peers as they age into older adulthood. This issue of “ageing-out” has been identified as problematic within other youth advocacy positions within mental health [2, 36]. Creating roles for youth peers to progress towards, helps to retain their knowledge and expertise within the system to continue to strengthen services [10] and helps to support these young people to advance in their career progression.

### Strengths and limitations

This paper describes research from a pilot study focused on the TAY program. Typically, realist methods apply a mixed method design, however, since peer-related program theories were developed later in the study through inductive analysis, we were not able to capture relevant quantitative data. Our findings are unique to this context and may not be characteristic of other youth peer programs. In addition, this research took place during the COVID-19 pandemic, therefore programming and study implementation occurred online. This may have influenced the findings and might not be a good reflection of how the program operates in-person. Finally, supervisor participation was limited to the initial focus group and one additional interview in the first round, therefore data from supervisors was limited and only partially informed theories. Placing additional focus on supervisor perspectives would be a good future direction within youth peer support research. However, this research profited from several strengths, including the benefits of combining a theory-driven and participatory approach and the significant insight gained from engaging a youth peer co-researcher in study oversight and implementation [16]. Participatory evaluation aligns well with peer support practice as it applies the knowledge from lived experience to inform research, elevating practicality of design, enriching the findings and increasing depth of insight. The participatory component was well-received by staff and enhanced feasibility of the realist component of the design (see [16] for more detail).

## Conclusion

This study applied a hybrid realist evaluation of the LOFT TAY peer services. The findings offer insight into the dynamics of how professional peer practice can support the personal development of the peers themselves and how programming can be intentionally designed to enhance these benefits. Peer practice holds great potential for the enhancement of the mental health system as well as to increase our understanding with respect to stigma. It is hoped that this study serves to enhance peer practice design as well as contributes to the development of mental health promotion interventions to support youth recovery and wellbeing more broadly.

## Declarations

### Research data

The data that supporting the findings of this study are available on request from the corresponding author. The data are not publicly available due to privacy or ethical restrictions.
